# The epithelioid gastrointestinal stromal tumor with pulmonary metastasis: A rare case report and literature review

**DOI:** 10.1097/MD.0000000000019346

**Published:** 2020-02-28

**Authors:** Dan Xu, Xuyong Lin, Xueshan Qiu

**Affiliations:** Department of Pathology, the First Affiliated Hospital and College of Basic Medical Sciences, China Medical University, Shenyang, China.

**Keywords:** epithelioid, gastrointestinal stromal tumor, mesothelioma, neuroendocrine carcinoma, pulmonary metastasis

## Abstract

**Rationale:**

Available literature states that the histological subtype of the gastrointestinal stromal tumor (GIST) with pulmonary metastasis is often spindle cell type. To our knowledge, this is the first report of the GIST with pulmonary metastasis of very uncommon epithelioid subtype.

**Patient concerns:**

We report a 63-year-old male presenting with the symptom of bloodstained sputum without obvious inducement. The patient had no chest pain, low back pain, fatigue, fever or night sweats symptoms.

**Diagnoses:**

Combined chest digital radiography and the history of the patient who presented with the colon GIST of the epithelioid subtype two years ago that the mass may be a metastasis tumor. Combined with morphological and immunohistochemical staining results, a pathological diagnosis of the GIST with pulmonary metastasis was considered.

**Interventions:**

Right lobectomy and partial upper lobectomy were performed.

**Outcomes:**

The patient had not experienced any noticeable symptom and recurrent tumors at 6 months follow-up.

**Lessons:**

We report a rare case of the GIST with pulmonary metastasis of epithelioid subtype. This case is of great significance to the pathologist's clinical work. For pathologists, if an epithelioid tumor in the lung is found, it is necessary to check whether the gastrointestinal tract also has the tumor, which may be an epithelioid GIST with pulmonary metastasis.

## 1 Introduction

Gastrointestinal stromal tumors (GISTs), the most common mesenchymal tissue tumors in the gastrointestinal tract, occur primarily in the elderly over 50 years old patients of either sex.^[1]^ GISTs are most commonly present in the stomach (50%–70%) and small intestine (25%–36%), but they also arise in the colon, rectum, appendix (5%–7% together), and esophagus (1%–3%).^[2]^ Although most GISTs are localized at presentation, up to half will recur locally, spread diffusely throughout the serosal surfaces of the abdomen.^[1]^ Approximately 10% to 30% of GISTs exhibit metastasis behavior.^[2]^ GISTs most often metastasize to the liver (50%–60%) and peritoneum (20%–43%), but they rarely metastasize outside the abdominal cavity, especially the bone and lung (10% together).^[3]^ Microscopically GISTs come in 3 different histological subtypes: pure spindle cells type (70%), pure epithelioid type (20%) or mixed morphology type (10%).

Authors checked PubMed and MEDLINE databases for the last 10 years searching for the epithelioid GIST with pulmonary metastasis. To our knowledge, this is the first report of the GIST with pulmonary metastasis of very uncommon epithelioid subtype. Herein, we report this case and review the literature with a special focus on the epithelioid subtype of GIST with metastasis.

## 2 Case report

**Figure 1:**
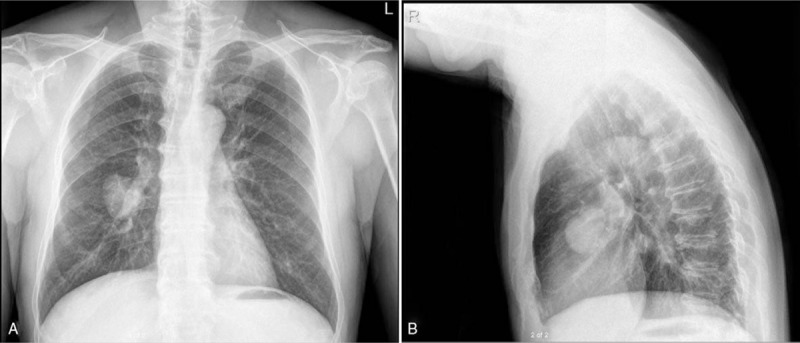
Images of chest forward (A) and lateral (B) digital radiography. It revealed bilateral thoracic symmetry and a mass with a diameter of about 5.2 cm was seen in the right middle lobe.

**Figure 2:**
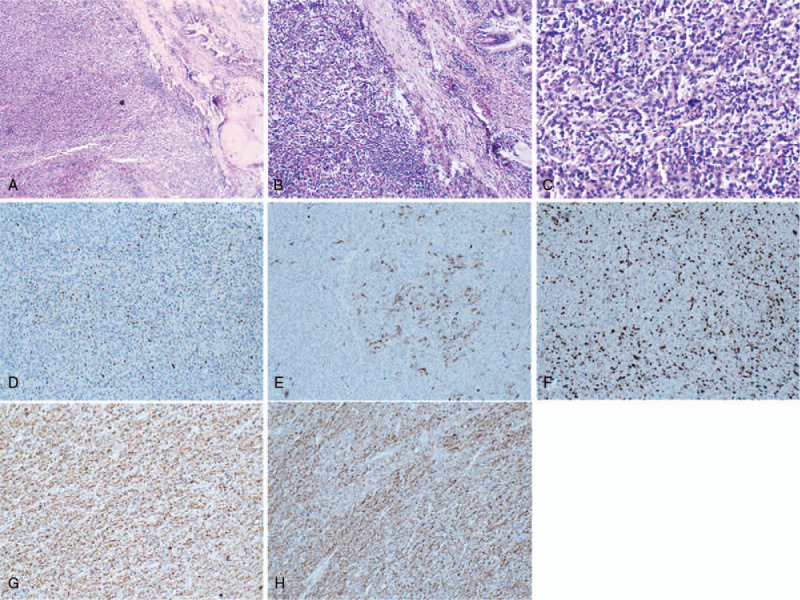
Histologic and immunohistologic feature of the mass. H&E showing epithelioid cells in the pulmonary tissue on low power (A, 40×) and high power (B: 100×, C: 400×). Immunohistochemical study showed positivity for P63 (D, 100×), SMA (E, 100×) and the Ki67 labeling index was estimated to be approximately 30% (F, 100x). Immunohistochemical study showed positivity for CD117 (G, 100×), Dog-1 (H, 100×).

In October 2018, a 63-year-old male presented with the symptom of bloodstained sputum, low volume and bright red color without obvious inducement. The patient had no chest pain, low back pain, fatigue, fever or night sweats symptoms. Chest digital radiography revealed a mass with a diameter of about 5.2 cm was observed in the right middle lobe (Fig. 1).Other clinical examinations were normal. The patient was treated with right lobectomy and partial upper lobectomy. On macroscopic examination, the tumor, approximately 4 cm in diameter, was located about 1.5 cm away from the hilar bronchus and 0.3 cm below the pleura. The boundary between the tumor and the surrounding tissue is clear. The tumor was sectioned and stained with H&E for further evaluation (Fig. 2A–C). Light microscopy revealed that tumor cells were epithelioid, round or oval. Immunohistochemical study showed positivity for P63 (scattered positive), smooth muscle actin (SMA) (multifocal positive) (Fig. 2D-E) and negativity for cytokeratin (CK), CD5/6, synaptophsin (Syn), thyroid transcription factor 1 (TTF-1), cytokeratin 7 (CK7), S-100, P40, CD56, leukocyte common antigen (LCA), Melan-A and SOX10. The Ki67 labeling index was estimated to be approximately 30% (Fig. 2F). So it excluded adenocarcinoma, and neuroendocrine carcinoma (NEC). However, the diagnosis of the tumor remained uncertain.

**Figure 3:**
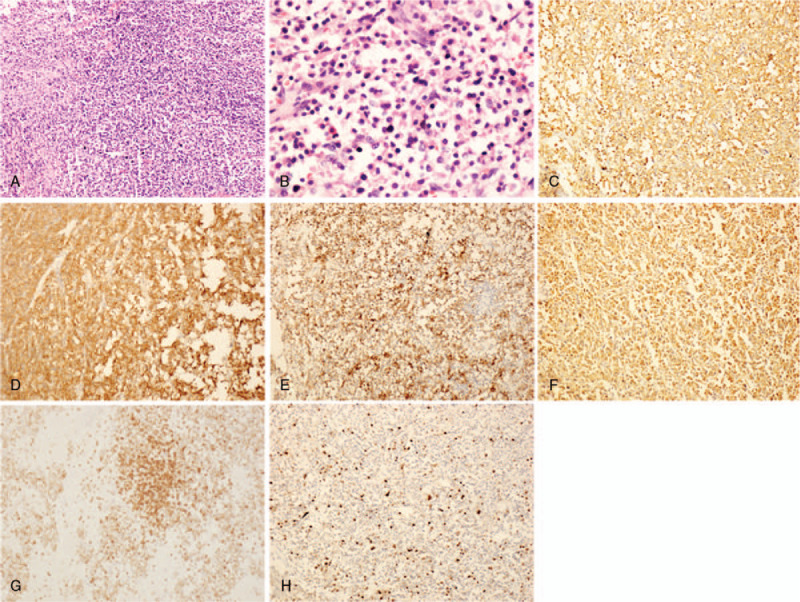
Histologic and immunohistologic feature of epithelioid gastrointestinal stromal tumor in the transverse colon. H&E showing that tumor cells are round or polygonal, the cytoplasm is rich in eosinophilic or bright on low power (A, 100×) and high power (B, 400×). Immunohistochemical study showed positivity for CD34 (C, 100×), CD117 (D, 100×), SMA (E, 100×), Vimentin (F, 100×), CD3 (G, 100×) and the Ki67 labeling index was estimated to be approximately 10% (H, 100×).

A review of the patient's history revealed that, in 2016, the patient presented with the colon GIST with epithelioid type. Microscopy revealed a proliferation of epithelioid cells in the lesion (Fig. 3A-B). Immunohistochemical study showed positivity for CD34,CD117,SMA,Vimentin (weakly positive),CD3 (few positive) and negativity for CK,CD56,Syn,TTF-1, Desmin,S-100,CD20,CDX-2 and Pax5. The Ki67 labeling index was estimated to be approximately 10% (Fig. 3C–H). So for this tumor in the lung, adenocarcinoma and NEC had been excluded, and we considered metastases to the pulmonary as the epithelioid GIST. Therefore, CD117 and Dog-1 staining were performed on lung tumor tissues, and the results showed that CD117 and Dog-1 were positive (Fig. 2G-H), effectively the diagnosis of the epithelioid GIST with pulmonary metastasis. The patient was followed up for 6 months without symptoms and residual/recurrent tumors.

## 3 Discussion

In general, GISTs display 3 different histological subtypes: pure spindle cells type (70%), pure epithelioid type (20%) or mixed morphology type (10%). The spindled, epithelioid and mixed morphology GISTs are reported to be significantly more common in the small intestine, stomach, and stomach, respectively.^[4,5]^ The growth of most GISTs is driven by oncogene mutations in either of two receptor tyrosine kinases: C-KIT (70%–80%) or platelet-derived growth factor receptor alpha (PDGFR-α) (8%–10%).^[4,6]^ No mutants for both genes of GISTs are wild type. Representative genotypes of epithelioid cell type GISTs are C-KIT gene mutants and *PDGFR-α* gene mutants. Approximately half of epithelioid GISTs are C-KIT gene mutations. In residual half of epithelioid cell type GISTs are *PDGFR-α* gene mutants.^[7]^

The frequency of C-KIT mutations in epithelioid GISTs may be lower than in spindle cell variants.^[8]^ C-KIT mutated GISTs with epithelioid/mixed phenotype are of significantly larger diameters and have significantly higher mitotic counts, high-risk categories and a short disease-free survival compared with their pure spindled type.^[4]^ These observations suggest that the spindled type represents the basic growth pattern in C-KIT mutant GISTs, whereas the epithelioid type may represent a secondary growth pattern.^[4]^ However, the differences in tumor diameter, mitotic counts, risk categories and disease-free survival between C-KIT mutated GISTs with pure spindled and epithelioid/mixed phenotypes are only observed in gastric GISTs, whereas there are no significant differences comparing tumors with different growth patterns from the small intestine and the colon.^[4]^ On the other hand, the predominance histopathologic phenotype of PDGFR-α mutated GISTs displays epithelioid or mixed (predominantly epithelioid) phenotypes.^[4]^ However, up-to-date studies did not reflect on an exact association of PDGFR-α mutant GISTs with a specific histopathologic phenotype.^[8]^

The epithelioid component displays unfavorable histological features (higher cellularity, higher mitotic activity and higher Ki67 index), and is associated with more aggressive clinical course. The presence of an epithelioid/mixed morphology component in GISTs is associated with malignant behavior in GISTs.^[4]^

### 3.1 Literature review

There are only 10 cases reported the epithelioid or mixed morphology type GISTs with metastasis, and the related clinical data of these 10 cases are summarized in the table (Table 1). The age of the 10 patients ranged from 13 to 70, with an average age of 48.7 years with a male to female ratio of 7:3. Two of ten cases the primary site of the tumor are the stomach. The size of the primary tumor ranges from 2 cm to 15 cm, with an average of 6.2 cm. Three cases report the histological of the metastasis tumor is composed of pure epithelioid cells. Others are mixed morphology type. The site of the metastasis tumor is in the abdominal cavity in only two cases and two is in the brain, two is in the bone, one is in the skin, one is in the supraclavicular lymph node, and one is in the lung (this case). There are very few cases of pure epithelioid or the mixed GISTs with metastasis, therefore, the pathologists must pay more attention to the diagnosis of the epithelioid/mixed GIST, especially metastasis to the rare location.

**Table 1:**
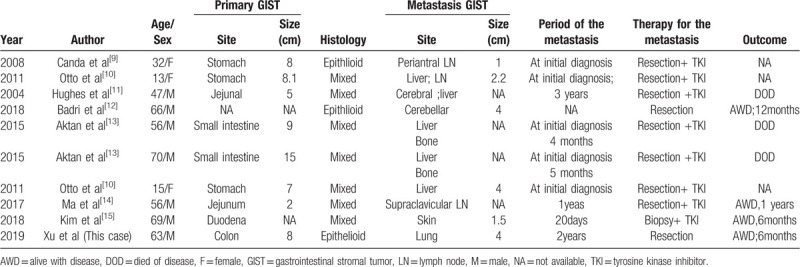
Related clinical data of the epithelioid or mixed morphology type GISTs with metastasis cases.

### 3.2 Diagnostic approaches

GISTs manifest a wide range of morphology, from spindle cell to epithelioid. The spindle subtype GISTs is composed of fusiform cells, rod-shaped or long fusiform nucleus. The epithelioid subtype GISTs is composed of uniformly rounded cells with abundant, clear to eosinophilic cytoplasm, and round or oval nuclei. The mixed subtype consists of cells with typical features of either spindle-shaped or epithelioid type.^[8]^ To diagnosis GISTs need immunocytochemical markers, as GISTs are not specific in the morphology.

CD117 and Dog-1 are immunocytochemical markers of GISTs characteristic in all cases.^[1]^ CD117 overexpression is the most sensitive and highly specific marker of stromal tumors detected in nearly 90% to 95% of cases. Although immunopositive for CD117 is the most sensitive marker of GISTs with the spindle type, approximately 82% of the epithelioid gastric GISTs show no signs of CD117 expression.^[5]^ In cases of epithelioid cell type GISTs with C-KIT gene mutations, CD117 is expressed strongly and diffusely. However, in the epithelioid cell type GISTs with *PDGFR-α* gene mutants, expression of CD117 is variable. Herein, diagnosis of GISTs with low or almost no CD117 expression may be difficult. Although CD117 and Dog-1 show a very similar expression pattern, Dog-1 may be more clearly positive in some *PDGFR-α* mutant GISTs, and CD117 may be more clearly positive than Dog-1 in other *PDGFR-α* mutant GISTs. In addition, Dog-1 and CD34 are expressed in most GISTs. Thus, Dog-1 immunohistochemical may be useful for diagnosis of the epithelioid GISTs with low or almost no CD117 expression.

Other markers such as CD34 and Ki67 are also representative but less specific. About half of epithelioid type GISTs can express CD34. However, the expression of CD34 varies greatly with the anatomical site. CD34 is almost universally positive in GISTs located in esophagus or rectum (95%–100%). CD34 is expressed in 80% to 88% of gastric GISTs. However, CD34-negative epithelioid-type gastric stromal tumors have been shown to be more aggressive.^[5]^ Ki-67 labeling index is variable from tumor to tumor. High Ki-67 labeling index and high mitotic counts might predict the high recurrence rate of the GISTs.^[7]^ SMA is expressed in only approximately 30% of patients, but is significantly more common in GISTs located in the stomach or small intestine. S-100 should be paid more attention to the diagnosis of GISTs, as it is present in only approximately 5% of patients and is especially common in epithelioid type tumors, the majority of which are more malignant.^[5]^ Expression of desmin, ALK, nuclear β-catenin and nuclear STAT6 is not usually detected.

### 3.3 Differential diagnosis

In clinical work, for the different type GISTs, it is necessary for the pathologist to differentiate them from many kinds of tumors, especially the GISTs metastasis to the rare location.

The spindle type GISTs must be distinguished from many kinds of tumors, such as fibromatosis (can be weakly CD117 positive), leiomyoma (beware of intermixed CD117-positive cells), leiomyosarcoma, schwannoma, malignant peripheral nerve sheath tumor, inflammatory myofibroblastic tumor, inflammatory fibroid polyp (may have PDGFR-α mutations), solitary fibrous tumor, synovial sarcoma, dedifferentiated liposarcoma, endometrial stromal sarcoma, sarcomatoid carcinoma.^[1]^

The epithelioid type GISTs must be distinguished from many kinds of tumors, such as epithelioid leiomyoma, NEC, malignant mesothelioma, metastatic melanoma and angiosarcoma.^[1]^

Cases of simultaneous occurrence of a GIST with a well-differentiated NEC have been reported at present, and CD117 is positive, so the pathologists need to pay more attention to the differentiation between GISTs and NEC.^[16,17]^ In the NEC, immunopositive for CK, CgA, and Syn.^[18]^ In the malignant mesothelioma, the immunocytochemical for CK5/6 and EMA are positive, but CD117 is not expression. About 30%-50% of malignant melanoma can express CD117, especially metastatic melanoma. Nevertheless, the expressions of Melan-A, HMB45, and S-100 were positive in malignant melanoma. In the pulmonary epithelioid angiosarcoma, immunopositive for CD31, CD34, and EGR.^[19]^

### 3.4 Treatment and prognostic

Surgical resection of the tumor and the use of tyrosine kinase inhibitors (TKI) drugs are the main methods for the treatment of GISTs.^[3]^ While treatment with TKI such as imatinib, sunitinib, and regorafenib are effective in controlling the unresectable disease.^[1]^ A surveillance, epidemiology, and end results analysis indicated that patients with GISTs with metastasis had a poorer prognosis than those without.^[20]^ Lung metastases and the epithelioid type seemed to be a poor prognostic factor in this patient with GISTs.^[21]^

## 4 Conclusion

We report a case of a 62-year-old man with the epithelioid GIST with pulmonary metastasis manifesting 32 months after surgical resection of the colon GIST for the first time. GISTs rarely metastasize to the lungs. Therefore, GISTs with pulmonary metastasis should be distinguished from various tumors. For pathologists, if an epithelioid tumor in the lung is found, it is necessary to check whether the gastrointestinal tract also has the tumor, which may be an epithelioid GIST with pulmonary metastasis. It is very rare that the epithelioid GISTs with metastasis. Therefore, pathologists must pay more attention to the diagnosis of the epithelioid/mixed GIST, especially metastasis to the rare location.

## Author contributions

**Conceptualization:** Dan Xu, Xuyong Lin, Xueshan Qiu.

**Investigation:** Dan Xu.

**Project administration:** Xueshan Qiu.

**Resources:** Dan Xu, Xuyong Lin.

**Writing – original draft:** Dan Xu.

**Writing – review & editing:** Dan Xu, Xuyong Lin, Xueshan Qiu.

Xueshan Qiu orcid: 0000-0002-7647-8110.
